# The association of diabetes mellitus and insulin treatment with expression of insulin-related proteins in breast tumors

**DOI:** 10.1186/s12885-018-4072-8

**Published:** 2018-02-27

**Authors:** Heleen K. Bronsveld, Marie L. De Bruin, Jelle Wesseling, Joyce Sanders, Ingrid Hofland, Vibeke Jensen, Marloes T. Bazelier, Bas ter Braak, Anthonius de Boer, Peter Vestergaard, Marjanka K. Schmidt

**Affiliations:** 1 Division of Molecular Pathology, The Netherlands Cancer Institute, Amsterdam, Netherlands; 2 Division of Pharmacoepidemiology & Clinical Pharmacology, Utrecht University, Utrecht, Netherlands; 3 Copenhagen Centre for Regulatory Science (CORS), University of Copenhagen, Copenhagen, Denmark; 4 Department of Pathology, The Netherlands Cancer Institute, Amsterdam, Netherlands; 5 Core Facility Molecular Pathology & Biobanking, The Netherlands Cancer Institute, Amsterdam, Netherlands; 6 Department of Pathology, Aarhus University Hospital THG, Aarhus, Denmark; 7 Division of Toxicology, Leiden Academic Centre for Drug Research, Leiden University, Leiden, Netherlands; 8 Departments of Clinical Medicine and Endocrinology, Aalborg University Hospital, Aalborg, Denmark

**Keywords:** Insulin, Insulin growth factor 1 receptor, Diabetes mellitus, Breast cancer, PI3K pathway, MAPK pathway, mTOR, Immunohistochemistry

## Abstract

**Background:**

The insulin receptor (INSR) and the insulin growth factor 1 receptor (IGF1R) play important roles in the etiology of both diabetes mellitus and breast cancer. We aimed to evaluate the expression of hormone and insulin-related proteins within or related to the PI3K and MAPK pathway in breast tumors of women with or without diabetes mellitus, treated with or without insulin (analogues).

**Methods:**

Immunohistochemistry was performed on tumor tissue of 312 women with invasive breast cancer, with or without pre-existing diabetes mellitus, diagnosed in 2000–2010, who were randomly selected from a Danish breast cancer cohort. Women with diabetes were 2:1 frequency matched by year of birth and age at breast cancer diagnosis to those without diabetes. Tumor Microarrays were successfully stained for p-ER, EGFR, p-ERK1/2, p-mTOR, and IGF1R, and scored by a breast pathologist. Associations of expression of these proteins with diabetes, insulin treatment (human insulin and insulin analogues) and other diabetes medication were evaluated by multivariable logistic regression adjusting for menopause and BMI; effect modification by menopausal status, BMI, and ER status was assessed using interactions terms.

**Results:**

We found no significant differences in expression of any of the proteins in breast tumors of women with (*n* = 211) and without diabetes (*n* = 101). Among women with diabetes, insulin use (*n* = 53) was significantly associated with higher tumor protein expression of IGF1R (OR = 2.36; 95%CI:1.02–5.52; *p* = 0.04) and p-mTOR (OR = 2.35; 95%CI:1.13–4.88; *p* = 0.02), especially among women treated with insulin analogues. Menopause seemed to modified the association between insulin and IGF1R expression (*p* = 0.07); the difference in IGF1R expression was only observed in tumors of premenopausal women (OR = 5.10; 95%CI:1.36–19.14; *p* = 0.02). We found no associations between other types of diabetes medication, such as metformin, and protein expression of the five proteins evaluated.

**Conclusions:**

In our study, breast tumors of women with pre-existing diabetes did not show an altered expression of selected PI3K/MAPK pathway-related proteins. We observed an association between insulin treatment and increased p-mTOR and IGF1R expression of breast tumors, especially in premenopausal women. This observation, if confirmed, might be clinically relevant since the use of IGF1R and mTOR inhibitors are currently investigated in clinical trials.

**Electronic supplementary material:**

The online version of this article (10.1186/s12885-018-4072-8) contains supplementary material, which is available to authorized users.

## Background

Approximately 10% of women diagnosed with breast cancer have pre-existing diabetes mellitus, which may affect their breast cancer progression, prognosis and treatment options [1–10]. Insulin (and the Insulin Growth Factor axes) appears to be an important factor linking diabetes and breast cancer [11–13]. In patients with diabetes, insulin metabolism is altered [14]. Type 2 diabetes is characterized by insulin resistance, and in earlier stages by hyperinsulinemia (high levels of endogenous insulin). Women with type 2 diabetes are usually treated with non-insulin antidiabetic drugs. If women with type 2 diabetes fail to respond to these glucose-lowering drugs over time, insulin (analogue) treatment is required. Patients with type 1 diabetes are insulin deficient and therefore rely on chronic treatment with insulin (analogues) [14].

Two important intracellular signaling pathways in the regulation of metabolism and cell growth are the phosphatidylinositol 3-kinase (PI3K-AKT) and mitogen-activated protein kinase (MAPK-ERK) [15]. Both pathways are involved in tumorigenesis and can be activated by insulin*.* Due to the high homology between the two isoforms of the insulin receptor (INSR-A and INSR-B) and the insulin growth factor 1 receptor (IGF1R), insulin can bind to INSR-A, INSR-B and IGF1R [16]. Insulin analogues are structurally transformed from human insulin and this may result in increased binding affinity towards the IGF1R [17, 18]. Phosphorylation of INSR-B, caused by insulin binding, preferentially induces metabolic signals via the PI3K pathway, while phosphorylation of INSR-A and IGF1R by insulin, predominantly leads to cell growth, and potentially tumor growth, via activation of the MAPK-ERK pathway [19]. One of the downstream proteins important for control of cell growth is mammalian target of rapamycin (mTOR), which can be activated by the PI3K or MAPK pathway via respectively extracellular signal-regulated kinases (ERK) or protein kinase B (AKT) [16].

In vitro and in vivo studies have shown that endogenous and exogenous insulin can stimulate tumor promotion via INSR and IGF1R. In vitro, insulin analogue stimulation increases proliferation of breast cancer cells due to enhanced IGF1R (and INSR) signaling, while exposure to human insulin showed low mitogenic potential [20]. Chronic treatment with insulin-like compounds (IGF1, insulin AspB10) with strong binding affinity towards the IGF1R, decreased the tumor latency time and showed increased MAPK-ERK signaling in a mammary gland mouse model, while insulin glargine and human insulin treatment did not significantly decrease the time for tumor development compared to the vehicle-treated mice [21].

Insulin might also stimulate tumor promotion via other receptors such as the estrogen receptor (ER) pathway. There is experimental support that insulin interacts with estrogens and might stimulate tumor growth via the ER pathway [13]. Moreover, estrogens enhance insulin signaling by increased expression and/or functional activity of proteins in the IR/ IGF1R pathway, which might results in enhanced proliferation [22]. Previous studies showed that IGF1R expression is higher in estrogen-dependent cell lines [19].

Little is known about diabetes/insulin exposure and protein signaling in tumors in the human setting. We hypothesized that tumors of patients with diabetes mellitus have higher expression of proteins in the insulin signaling pathway, especially among those treated with insulin and/or insulin analogues. Specifically, we aimed to evaluate the expression of (downstream activated) proteins within or related to the PI3K and MAPK pathways.

## Methods

### Study design, patient selection and data collection

**Fig. 1:**
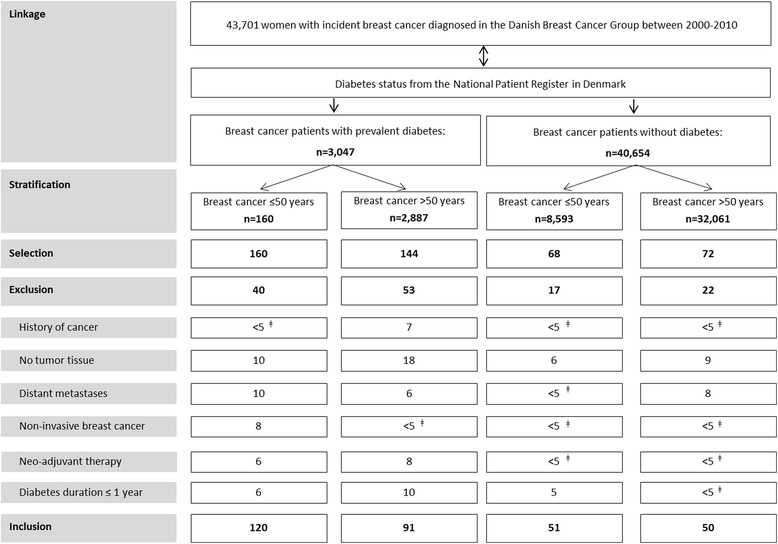
Flow chart of patient identification and selection. Stratified by age at breast cancer diagnosis (≤50 and > 50 years), women with diabetes were 2:1 frequency-matched on year of birth and age at breast cancer diagnosis (both in 10-year categories) to women without diabetes, to select ~ 300 patients with tumor tissue available. ǂ Exact numbers < 5 cannot be shown according to regulations of Statistics Denmark

We conducted a cross-sectional study with a target population of ~ 300 breast cancer patients, randomly selected from an existing nationwide hospital-based cohort set up by the Danish Breast Cancer Cooperative Group (DBCG), of women with primary breast cancer (*N* = 43,701) diagnosed between 2000 and 2010 [23]. Details on patient selection and methods of data collection have been described previously [24]. In short, the selected women included breast cancer patients with pre-existing diabetes (exposed) and without diabetes (non-exposed) sampled as follows: we frequently matched by year of birth and age at diagnosis (both in 10-year categories)a random sample of women with diabetes (in strata of age ≤ 50 and > 50 years at breast cancer diagnosis) to women without diabetes (Fig. 1) [24]. To allow analyses of insulin treatment, we included two times as many women with diabetes as women without diabetes. We excluded patients with a history of other cancers, non-invasive or metastasized breast cancer, those treated with neo-adjuvant therapy, patients with diabetes diagnosed ≤1 year prior to their breast cancer diagnosis, and patients with no or insufficient tumor tissue. We obtained data from the DBCG database, linkage with the National Patient Register in Denmark, linkage with the Danish Register of Medicinal Products Statistics, and additional information on height, weight and Body Mass Index (BMI) prior to breast cancer diagnosis from electronic medical records [24]. The study protocol was approved by the Science Ethics Committee of the Region Midtjylland in Denmark (M-20110198).

### Diabetes treatment classification

As described also previously [24], we classified diabetes status based on medical diagnosis from the Danish National Patient Register; and diabetes duration as time from age of diabetes diagnosis till age of breast cancer diagnosis. We assigned women with diabetes to a treatment group if at least 2 prescriptions of an antidiabetic drug were prescribed in the period up to one year prior to breast cancer diagnosis. We defined exposure time as time from age of start of the antidiabetic drug till age of breast cancer diagnosis. We classified women with diabetes treated with insulin only as type 1 diabetes, if they had a recorded diagnosis of type 1 diabetes (*n* = 21), or if a medical code was missing but they were under age 30 years at diabetes diagnosis (*n* = 4); all other women with diabetes were classified as type 2. We compared women with diabetes who had a history of treatment with: insulin (human insulin and/or insulin analogues) vs. never treated with insulin; insulin with non-insulin antidiabetic drugs vs. insulin only; insulin analogues vs. human insulin only; any antidiabetic medication vs. diet and exercise only; metformin vs. no metformin. In the analyses comparing insulin and concomitant non-insulin antidiabetic drug users vs. insulin only users, we excluded women who were treated with diet and exercise only. In the analyses comparing metformin vs. no metformin users, we only included women who had a history of treatment with non-insulin antidiabetic drugs.

### Tumor block collection and immunohistochemical (IHC) analyses

Formalin-fixed, paraffin embedded tissue samples of the primary tumors were retrieved from different pathology departments in Denmark. Morphology, grade, tumor size, number of positive lymph nodes and clinical tumor subtype, immunohistochemically defined by ER, PR, and human epidermal growth factor receptor 2 (HER2) status, were available from central pathology review [24]. All formalin-fixed, paraffin embedded tumor blocks of the primary tumor of each patient were collected, sectioned and hematoxylin and eosin (HE) stained. Two cores of 2 mm were taken from the most representative tumor block of each patient for constructing duplicate Tissue Micro Arrays (TMAs), with one core of each patient on both TMAs. We chose hormone and insulin-related proteins within or related to pathways of interest (MAPK and PI3K) that were previously stained in the Netherlands Cancer Institute and/or reported in scientific articles with IHC application: p-ER, epidermal growth factor receptor (EGFR), INSR, IGF1R, p-ERK1/2, p-mTOR, phospho-ribosomal protein S6 kinase beta-1 (p-P70S6), and p-AKT. Antibodies for INSR, p-P70S6K, and p-AKT did not show sufficient validity and reliability on human breast tissue; staining was weak or showed variations in staining pattern. Varying dilutions of these antibodies and/or staining procedure (manual versus automated) did not lead to improvement. The antibodies for p-ER, EGFR, IGF1R, p-ERK1/2, and p-mTOR, were all developed and validated on human breast tissue by the Core Facility Molecular Pathology & Biobanking (CFMPB) of the Netherlands Cancer Institute. For each antibody a positive control was included.

Immunohistochemistry was performed on a BenchMark Ultra autostainer (Ventana Medical Systems). Briefly, 3 μm paraffin sections of TMAs were cut using a microtome, these sections were heated at 75 °C for 28 min, and deparaffinised in the autostainer with ‘EZ prep’ solution (Ventana Medical Systems). Heat-induced antigen retrieval was carried out using Cell Conditioning 1 (CC1, Ventana Medical Systems) for respectively 36 (p-mTOR), 64 (p-ERK1/2, EGFR, IGF1R) and 92 (p-ER) minutes at 95 °C. Primary antibody incubation times were 16 min (EGFR, IGF1R), 32 min (p-ER) and 1 h (p-mTOR, p-ERK1/2). Details of the used antibodies, dilutions and localization of staining are summarized in Additional file 1: Table S1. Bound antibody was detected using the UltraView Universal DAB Detection Kit (Ventana Medical Systems). Slides were counterstained with Hematoxylin and Bluing Reagent (Ventana Medical Systems).

**Fig. 2:**
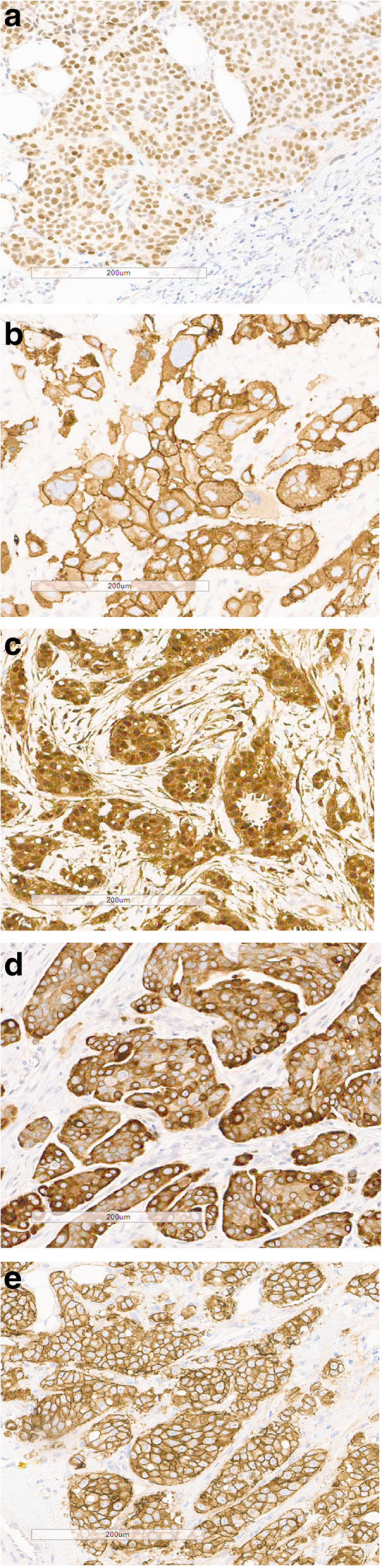
Patterns of immunohistochemical protein expression. **a** p-ER nuclear staining (70%), (**b**). EGFR membrane staining (100%), (**c**) p-ERK1/2 nuclear/cytoplasmic staining (100%), (**d**) p-mTOR cytoplasmic staining (100%), (**e**). IGFR strong complete membrane/cytoplasmic staining (≥10%)

Scoring of the IHC staining was performed by a breast pathologist (JS). The percentages of stained tumor cells were assessed for P-ER, EGFR, p-ERK1/2 and p-mTOR using a 10% step scale (0–100%). However, only the percentages of tumor cells stained with moderate to strong intensity were taken into account. The low intensity staining was very weak and therefore it was unclear whether the actual tumor cells or the background was stained. We aimed to create a binary variable for a positive and negative staining. The cut-off for ER, PR and HER2 status is clear from daily practice (http://www.oncoline.nl/borstkanker) (< 10% is negative)). However, for none of the other markers of interest there was a clinically defined cut-off available and we had to define cut-off values based on available literature, median expression levels (Additional file 1: Table S2) and advice of an experienced breast pathologist (JW), before association analyses were carried out. P-mTOR was considered positive if cytoplasmic staining was present in ≥40% of the cells. For p-ER and EGFR was decided on a 10% cut-off for a positive nuclear and respectively membrane staining [25, 26]. P-ERK1/2 was considered positive if either nuclear or cytoplasmic staining was present in ≥10% of the cells [27]. IGF1R expression was scored negative for no staining or weak partial membrane or cytoplasmic staining and was scored positive if ≥10% of the tumor cells had a moderate or strong complete membrane or cytoplasmic staining [28, 29]. Figure 2 gives an overview of protein expression patterns of all proteins that were stained with moderate to strong staining. For all markers, discordant results between the two cores of each patient were revised and in case of a difference, the highest score was used for the analyses. If one core failed, the value of the remaining core was included in the analysis. Only the invasive part of the tumor, as judged by the pathologist, was considered when scoring the staining. When no (invasive) tumor cells were available, the result of the staining was coded as a missing value. Reporting recommendations for Tumor Marker Prognostic Studies (REMARK) have been followed (Additional file 2: document 1).

### Statistical analyses

We hypothesized that diabetes, and in particular insulin use, would be associated with high(er) expression of IGF1R/EGFR and downstream activated proteins p-ERK1/2 and p-mTOR. Our primary analysis was therefore to test whether the expression of these proteins in breast tumors was dependent on diabetes status or insulin use, the latter analysis was restricted to women with diabetes only. We analyzed these markers as a binary factor in a multivariable logistic regression model, using the cut-off value as specified above. For significant findings of continuously scored markers, the proportion of positively stained tumor cells were analyzed as a continuous factor using a zero-inflated binomial (ZIB) model, as the data were not normally distributed. The ZIB model consists of a count component (negative binomial) and a binary component (logistic) and gives parameter estimates for both [30]. We did not perform this analysis for IGF1R since we did not continuously score the proportion of positively stained tumor for IGF1R.

Potential covariates, i.e. year of breast cancer diagnoses, age, menopausal status, BMI and diabetes duration, were individually added to the model and were only included if the beta-estimate for diabetes or insulin changed > 10%. Menopause and BMI changed the beta for diabetes with > 10% in the analyses of p-ER, EGFR, p-mTOR and IGF1R, and the beta for insulin in the analyses of p-ER, EGFR and p-ERK1/2. Therefore, for simplicity and consistency of between marker comparisons, all models were adjusted for menopause and BMI. Though we had previously shown that grade, tumor size, and positive lymph node status were not associated with our outcome of interest [24], we ran full models including these three variables to confirm that our conclusions based on statistically significant findings remained unchanged. For patients with unknown menopausal status (*n* = 5), age over 52 years [31] was used as a proxy for postmenopausal status. As previously described [24], we imputed missing values for BMI (*n* = 93) using Multivariate Imputations by Chained Equations (MICE) [32] in R studio with a predictive mean matching regression model for each analyzed dataset, imputing variables with ascending number of missing values; number of imputations = 10, number of iterations = 25; see (Additional file 1: Table S3). We assumed that data was missing at random and could be imputed because of correlations with other variables (e.g. smoking, alcohol, cardiovascular disease, microvascular disease, income, education, diabetes type, diabetes duration, height, weight).

Modifications of the associations between diabetes status/insulin use and proteins of interest by menopausal status, BMI and ER status were assessed using interactions terms. To exclude potential bias by the inclusion of patients with type 1 diabetes we performed a sensitivity analysis comparing women with type 2 diabetes only to women without diabetes. We also tested for heterogeneity of expression of proteins between tumors of type 1 and type 2 diabetes patients using insulin. A *p*-value of < 0.05 was defined as statistically significant. SAS Enterprise guide 4.2 for Windows was used for all analyses.

## Results

The cross-sectional study consisted of 211 women with diabetes and 101 women without diabetes, all diagnosed with breast cancer and with tumor tissue available (Fig. 1). Patient and breast cancer characteristics at diagnosis have been published in detail previously and have been summarized in Additional file 1: Table S4 and S5. Most women with diabetes were categorized as type 2 (88.2%). Immunohistochemistry could be evaluated in 93–96% of breast tumors, dependent on each marker (Additional file 1: Table S2). In the evaluated tumors, positive protein expression was found in 47% for p-ER, 9% for EGFR, 55% for p-ERK1/2, 59% for p-mTOR and 73% for IGF1R, respectively (Additional file 1: Table S2).

**Fig. 3:**
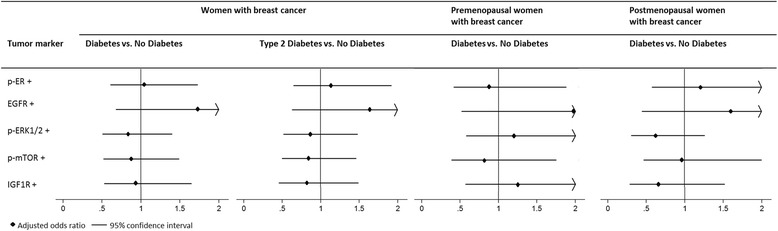
Odds ratios for tumor protein expression status of women with diabetes compared to women without diabetes using logistic regression. Odds ratios were adjusted for menopause and BMI. For details of the analyses see Table 1 and Additional file 1: Table S7

We found no significant differences in tumor expression of any of the selected proteins between women with and without diabetes (Fig. 3, Table 1 and Additional file 1: Table S6). Exclusion of women with type 1 diabetes gave similar results (Fig. 3, Additional file 1: Table S6 and S7). We found no effect modification of any of the proteins by menopause, ER status or BMI). However, because we previously found that menopause modified the association between diabetes and breast cancer subtype, we also presented the results stratified for menopause. After stratification for menopause (Fig. 3, Additional file 1: Table S6 and S7), we noticed that the direction of the effects of diabetes on p-ER, p-ERK1/2 and IGF1R differed in pre- and postmenopausal women.

**Table 1:** Odds ratios for tumor protein expression status of women with diabetes compared to women without diabetes

Women with breast cancer				
	Independent variable of exposure			
	Diabetes vs. No Diabetes		Diabetes vs. No Diabetes	
Dependent variable^a^	crude OR (95% CI)	P	adjusted OR^b^ (95% CI)	P
p-ER +	0.84 (0.51–1.37)	0.48	1.03 (0.61–1.73)	0.92
EGFR +	1.44 (0.59–3.52)	0.43	1.72 (0.68–4.33)	0.25
p-ERK 1/2 +	0.84 (0.52–1.37)	0.48	0.84 (0.51–1.40)	0.51
p-mTOR +	0.81 (0.49–1.33)	0.40	0.88 (0.52–1.49)	0.64
IGF1R +	0.90 (0.52–1.56)	0.70	0.94 (0.53–1.65)	0.82

^a^Logistic regression for tumor IHC marker as the dependent variable, with a negative staining of the tumor marker as reference category. ^b^Adjusted for menopause (pre/post) at breast cancer diagnosis and BMI closest measure prior to breast cancer diagnosis (continuous). Women with diabetes were matched on age at breast cancer diagnosis to women without diabetes. *p-ER* Phosphorylated estrogen receptor, *EGFR* Epidermal growth = factor receptor, *p-ERK* Phosphorylated extracellular signal-regulated kinase, *p-mTOR* Phosphorylated mechanistic target of rapamycin, *IGF1R* Insulin growth factor 1 receptor, *OR* Odds Ratio, *CI* Confidence Interval

**Fig. 4:**
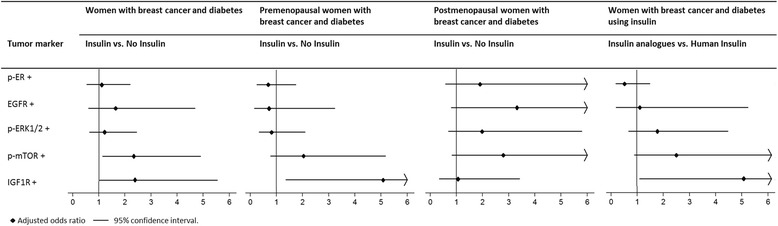
Odds ratios for tumor protein expression status of women with diabetes treated with insulin compared to women not treated with insulin using logistic regression. Odds ratios were adjusted for menopause and BMI. For details of the analyses see Table 2 and Additional file 1: Table S10

Twenty-five percent (*n* = 53) of the women with diabetes were treated with insulin, of which 18 combined insulin with non-insulin antidiabetic drugs (Additional file 1: Table S5). Among the insulin users, 28 were treated with human insulin only and 25 used insulin analogues with (*n* = 22) or without human insulin (*n* = 3). The non-insulin users (75%, *n* = 158) were treated with non-insulin antidiabetic drugs (*n* = 74) or diabetes was controlled by diet and exercise only (*n* = 84). Any insulin use was significantly associated with higher expression of IGF1R (OR = 2.36; 95%CI:1.02–5.52; *p* = 0.04) and p-mTOR (OR = 2.35; 95%CI:1.13–4.88; *p* = 0.02; Fig. 4, Table 2 and Additional file 1: Table S8) in breast tumors. The ORs for IGF1R and p-mTOR additionally adjusted for grade, tumor size and positive lymph node status were respectively 2.32 (95%CI:1.02–5.30; *p* = 0.05) and 2.58 (95%CI:0.97–6.81; *p* = 0.06). Additional analyses including the proportion of positively stained tumor cells as a continuous factor (using the ZIB model) gave similar results (data not shown); e.g. in the analyses for p-mTOR, the binary components explained most of the difference (estimate = − 1.21, *p* = 0.02), while the count component did not add much (estimate = 0.03, *p* = 0.80). Therefore, the logistic analyses were appropriate and using the data continuously did not improve the model. Expression of IGF1R significantly differed between insulin analogues users (*n* = 28) and users of human insulin only (*n* = 25) (Fig. 4, Additional file 1: Table S8 and S10). Insulin analogue users more often developed tumors that expressed IGF1R compared to human insulin only users (OR = 4.94; 95%CI:1.11–21.92; *p* = 0.04). The OR for p-mTOR was also higher among insulin analogue users, but not significantly different (OR = 2.46; 95%CI:0.91–6.63; *p* = 0.08) (Fig. 4, Additional file 1: Table S8 and S10).

**Table 2:** Odds ratios for tumor protein expression status of women with diabetes treated with insulin compared to women not treated with insulin

Women with breast cancer and diabetes				
	Independent variable of exposure			
	Insulin^b^ vs. No Insulin^c^		Insulin^b^ vs. No Insulin^c^	
Dependent variable^a^	crude OR (95% CI)	P	adjusted OR^d^(95% CI)	P
p-ER +	1.13 (0.38–2.19)	0.73	1.08 (0.53–2.19)	0.82
EGFR +	1.84 (0.69–4.91)	0.22	1.67 (0.60–4.67)	0.33
p-ERK 1/2 +	1.31 (0.68–2.53)	0.42	1.24 (0.63–2.44)	0.54
p-mTOR +	**2.41 (1.18–4.93)**	**0.02**	**2.35 (1.13–4.88)**	**0.02**
IGF1R +	**2.47 (1.07–5.67)**	**0.03**	**2.36 (1.02–5.52)**	**0.04**

^a^Logistic regression for tumor IHC marker as the dependent variable, with a negative staining of the tumor marker as reference category. ^b^Women with diabetes treated with insulin (analogues) regardless the use of concomitant noninsulin antidiabetic drugs. ^c^Women with diabetes treated only with diet and exercise and users of noninsulin antidiabetic drugs only. ^d^Adjusted for menopause (pre/post) at breast cancer diagnosis and BMI closest measure prior to breast cancer diagnosis (continuous). *p-ER* Phosphorylated estrogen receptor, *EGFR* Epidermal growth = factor receptor, *p-ERK* Phosphorylated extracellular signal-regulated kinase, *p-mTOR* Phosphorylated mechanistic target of rapamycin, *IGF1R* Insulin growth factor 1 receptor, *OR* Odds Ratio, *CI* Confidence Interval

Menopause seemed to modify the association between insulin and IGF1R expression (*p* = 0.07) and the difference in IGF1R expression between tumors of insulin and non-insulin users was only observed among premenopausal women with diabetes (OR = 5.10; 95%CI:1.36–19.14; *p* = 0.02; Fig. 4, Additional file 1: Table S9 and S10). The same results were found when we additionally adjusted for grade, tumor size and positive lymph node status (OR = 5.08; 95%CI:1.24–20.86; *p* = 0.02). We found no significant interaction between insulin use and ER status (*p* ≥ 0.15) or BMI (*p* ≥ 0.20). However, because the origin of the present breast cancer subtype classification is largely based on ER status; we confirmed that results were similar if analyses were stratified by ER-status (Additional ile 1: Table S9 and S10). Adjustment for ER status in the multivariable model did also not materially change the estimates, but adjustment for breast cancer subtype (Luminal A/Luminal B/HER2-positive/triple negative) led to slightly stronger associations of insulin with IGF1R (OR = 2.78; 95%CI:1.09–7.09; *p* = 0.03) and p-mTOR (OR = 3.42; 95%CI:1.43–8.17; *p* = 0.006), with more expression of IGF1R and p-mTOR in triple negative, and less expression in HER2 positive tumors. We found no significant heterogeneity between tumor expression of the proteins of interest between diabetes type 1 and type 2 insulin users, except for p-ER (type 1 vs. type 2: OR = 0.28; 95%CI:0.08–0.95; *p* = 0.04) (Additional file 1: Table S8 and S10), but after adjustment for menopause and BMI this difference was non-significant.

We observed no statistically significant differences between expression of any of the proteins among tumors of women with diabetes treated with a combination of insulin and non-insulin-antidiabetic drugs compared to insulin-only users, nor did we find differences between tumors of women with diabetes treated with any diabetes medication compared to women with diabetes treated with diet and exercise only. In our study, 69% (*n* = 51) of the women treated with non-insulin antidiabetic drugs only were treated with metformin (Additional file 1: Table S5). We did not find a significant decreased effect of p-mTOR activation in tumors of metformin users (*n* = 51) compared to non-insulin antidiabetic drug users not treated with metformin (*n* = 23) (OR = 0.57; 95%CI:0.21–1.56; *p* = 0.27), nor did we find differences in any of the other proteins.

## Discussion

We found no strong evidence that p-ER, EGFR, p-ERK1/2, p-mTOR, or IGF1R are differently expressed in breast tumors of women with and without diabetes. We showed that insulin treatment is associated with higher IGF1R and p-mTOR tumor expression in women with diabetes. Among insulin users, IGF1R was significantly more often expressed in tumors of women treated with insulin analogues compared to women treated with human insulin only. We found no strong evidence for an association between other types of diabetes medication, such as metformin, and any of the proteins that were assessed.

Insulin treatment was only associated with IGF1R expression in tumors of premenopausal women with diabetes. We have indications that this difference between pre- and postmenopausal women is related to hormonal difference related to tumor subtype because previously, we found that premenopausal women with breast cancer and diabetes more often develop tumors that do not express hormonal receptors (especially among women with type 1 diabetes) [24]. In ER-negative tumors we see a tendency towards higher IGF1R expression (Additional file 1: Table S10) and in multiple regression analyses adjusting for breast cancer subtype it was confirmed that IGF1R was more expressed in triple negative tumors. This might indicate that in women with tumors not expressing hormone receptors, the IGF1R signaling pathway might be an alternative way of breast cancer development, since this type of tumor is not dependent on the common ER/PR-signaling. We also found that ER is more often phosphorylated in women using insulin with type 2 diabetes compared to type 1 diabetes, which is in line with our previous findings that type 2 diabetes insulin users had more often ER-positive tumors compared to women with type 1 diabetes [24]. It has been suggested that phosphorylation of ER (at Ser^118^) indicates that the ER signaling pathway in breast cancer is intact and that it is correlated with responsiveness of breast cancer to tamoxifen [26]. We did not find an association between metformin use and p-mTOR or any other of the examined proteins, while it has been suggested that metformin can decrease INSR and IGF1R signaling and can inhibit mTOR [33].

It should be noted that the specific proteins we investigated, especially IGF1R, are involved in signaling pathways that interfere with other growth factor receptor pathways such as ER, PR and EGFR. Therefore, expression of these proteins should be interpreted in the context of breast cancer subtype [29]. In our study, adjustment for ER status did not materially change the results and adjustment for breast cancer subtype led to slightly stronger associations of insulin with IGF1R and p-mTOR. We found no interaction between insulin use and ER status and we confirmed that results were similar when analyzed by ER-status.

As far as we know, two previous studies in humans, with small sample size (*n* = 39–40), examined protein or gene expression of the IGF1, IGF2, IGFBP3, INSR, IGF1R and downstream targets IRS1, IRS2 and mTOR in women with or without type 2 diabetes [34, 35]. Both studies found no association between these proteins and diabetes, which is in line with our findings, except for IGF1R protein expression that was found to be significantly higher in women with diabetes [35]**.** Since these studies had no data on insulin treatment among women with diabetes and the duration and severity of diabetes might differ between studies, it is hard to explain differences between their results and ours. In vitro studies have shown that the PI3K signaling pathway [36–39] and the MAPK pathway [36, 37] are significantly upregulated after stimulation of insulin analogues compared to human insulin. In mammary gland tumors of mice, expression of IR, IGF1R and p-AKT was significantly higher in insulin or insulin analogues-treated compared to vehicle-treated mice, while expression of p-ERK was only increased among tumors of mice treated with insulin analogues [21]. Our results suggest that treatment with insulin and insulin analogues increases signaling via mTOR. However, since the relationship between IGF1R expression and downstream activation of the m-TOR pathway is not consistent in pre- and postmenopausal women, these findings suggest that insulin use may increase p-mTOR independently of increased IGF1R expression. Since we could not stain p-AKT and the PI3K and MAPK pathway interacts with many other proteins/pathways, we can only speculate about the actual signaling pathways involved. .

A strength of our study is that data were collected from a randomly selected group of women, based on data of comprehensive Danish biobanks, included medication history at least 5 years prior to breast cancer diagnosis from prescription records. We expect that this resulted in a patient selection that was minimally affected by several forms of bias, such as survival, selection or ascertainment bias, as discussed previously [24]. All stainings were validated and performed in one centre and scored by the same experienced breast pathologist, to prevent inter-laboratory and inter-observer variability [40, 41] and to assure quality and completeness of the data. We scored staining intensity and percentage positive tumor cells, and used the continuous expression data to validate our binary analyses. Median expression levels in our study corresponded with median expression levels and cut-offs used in previous studies examining these proteins [25–29]. The percentages of positive expression for p-ER, EGFR, p-ERK1/2, p-mTOR and IGF1R were also in line with previous published data, using similar population selection, IHC methods, and assessment criteria in primary invasive breast tumors [26, 27, 29, 35, 42]. Additionally, effects estimates were adjusted for potential confounders and analyzed for potential effect modifiers and are therefore less likely to be distorted by the presence of other factors.

We had limited power to study differences of tumor protein expression among insulin users in women with type 1 and type 2 diabetes and between insulin analogues users and human insulin only users. Moreover, the 95% confidence intervals around the presented estimates are wide and might be subject to change. Therefore we have to interpret both our positive and null results with caution and we recommend others to confirm our findings in a larger dataset.

Another important point that we need to address is that in patients with type 2 diabetes treatment choices for the management of diabetes are based on the severity of the disease. Although diabetes duration did not change the beta-estimate for diabetes or insulin with > 10% for any of the proteins, we had no power to investigate duration/dose of insulin exposure and the effect on tumor protein expression. There is a (small) chance that our findings are subject to confounding by indication; severe diabetes and not insulin is related with increased p-mTOR and IGF1R expression. Furthermore, many other factors, such as the nutritional/metabolic status [43, 44] and endogenous insulin levels [45–47] of the patients, which are interrelated with the severity of diabetes and diabetes treatment, might influence mTOR signaling. Unfortunately, we could not adjust for this in the analysis since we had no information on the metabolic status and c-peptide levels of the patients. Altogether, this makes it extremely hard to disentangle the true association between insulin treatment and IGF1R/mTOR expression.

Although the majority of insulin users had prescriptions of insulin over several years prior to breast cancer diagnosis (mean: 8.4 years), we cannot guarantee the sequence of events (insulin exposure and subsequent tumor promotion) because of the potential lag time in the detection of the tumor. However, tumor size (an important factor for detection) was not related to diabetes status or insulin exposure, so it is unlikely that the associations we observed were due to reverse causation.

Due to the small frequencies of tumors that expressed EGFR, we could not interpret the results of this receptor. Unfortunately, antibodies targeting staining of the INSR and other proteins in the PI3K and MAPK pathway (such as AKT and p-P70S6K) did not work on our series of human breast tumor samples, as explained in the methods. Furthermore, we could not examine the phosphorylation state of the INSR compared to the IGF1R since there is only a non-specific p-INSR/p-IGF1R antibody available yet. BMI was collected from the medical records of the patients and were incomplete. However, since we had extensive data on variables correlated with BMI, we were able to impute missing values using multiple imputations. Although the ratios for observed and imputed BMI were similar, BMI could still be misclassified for some patients. At last, we considered that embedding and storage of tissue blocks may have been different between pathology laboratories, and this could have affected the results of the staining. However, this would only have confounded the analyses if diabetes status or insulin use would have been differentially distributed between laboratories or years of diagnosis, and this was not the case (Additional file 3: Figure S1).

## Conclusions

We found that insulin treatment in women with diabetes is associated with p-mTOR tumor expression, and in premenopausal women with IGF1R tumor expression. However, more research is needed to confirm our findings and to explore the role of insulin signaling in breast cancer initiation and/or promotion in patients with diabetes, especially among those using insulin or insulin analogues. This observation, if confirmed, might be clinically relevant since currently the use of IGF1R and mTOR inhibitors are investigated among breast cancer patients in clinical trials [19, 48, 49]. IGF1R and mTOR inhibitors might interfere with glucose metabolism and iatrogenic diabetes has been reported as side effect of IGF1R inhibitors. Therefore monitoring for hyperglycemia and dyslipidemia is important and the use of these inhibitors might be limited in patients with diabetes [50, 51].

## Additional files

Additional file 1: Table S1. Antibodies used for immunohistochemical assays. **Table S2** Overview of the number of positively stained and unevaluable immunohistochemical markers; with for the evaluable cores the median percent of tumor cells with moderate to strong protein expression. **Table S3.** Average Body Mass Index of breast cancer patients in subgroups of menopausal status, in the ten imputed datasets (% (n)). **Table S4.** Characteristics of breast cancer patients with and without diabetes and of insulin and non-insulin users. **Table S5.** Patient characteristics and medication use among women with type 1 and type 2 diabetes. **Table S6.** Numbers and proportions of tumor protein expression status of women with diabetes, type 2 diabetes and without diabetes in all women and in subgroups of menopausal status. **Table S7.** Odds ratios for tumor protein expression status of women with type 2 diabetes compared to women without diabetes, and of women with and without diabetes in subgroups of menopausal status. **Table S8.** Number and proportion of tumor protein expression status of women with type 1 and type 2 diabetes treated with insulin (human and analogues) and without insulin. **Table S9.** Number and proportion of tumor protein expression status of women with diabetes treated with insulin and without insulin in subgroups of menopausal status and ER tumor status. **Table S10.** Odds ratios for tumor protein expression status of women with diabetes; treated with insulin analogues compared to women treated with human insulin; type 1 compared to type 2 insulin users; treated with insulin compared to women not treated with insulin in subgroups of menopausal status and in subgroups of ER tumor status. (DOCX 88 kb)

Additional file 2: Document 1. REMARK checklist. (PDF 68 kb)

Additional file 3: Figure S1. Diabetes status stratified by pathology laboratories (%). (PNG 38 kb)

## Abbreviations

AKT: Protein kinase B
BMI: Body mass index
CFMPB: Core Facility Molecular Pathology & Biobanking
CI: Confidence interval
DBCG: Danish Breast Cancer Cooperative Group
EGFR: Epidermal growth factor receptor
ERK: Extracellular signal-regulated kinases
HE: Hematoxylin and eosin
HER2: Human epidermal growth factor receptor 2
IGF1R: Insulin growth factor 1 receptor
IHC: Immunohistochemical
INSR: Insulin receptor
MAPK: Mitogen-activated protein kinase
MICE: Multivariate Imputations by Chained Equations
mTOR: Mammalian target of rapamycin
ER: Oestrogen receptor
P-: Phosphorylated
PI3K: Phosphatidylinositol 3-kinase
PR: Progesterone receptor
P70S6: Ribosomal protein S6 kinase beta-1
TMA: Tissue Micro Array
ZIB: Zero-inflated binomial

## References

[1] Erickson K, Patterson RE, Flatt SW, Natarajan L, Parker BA, Heath DD, Laughlin GA, Saquib N, Rock CL, Pierce JP (2011). Clinically defined type 2 diabetes mellitus and prognosis in early-stage breast cancer. Journal of clinical oncology : official journal of the American Society of Clinical Oncology.

[2] Gillespie EF, Sorbero ME, Hanauer DA, Sabel MS, Herrmann EJ, Weiser LJ, Jagielski CH, Griggs JJ (2010). Obesity and angiolymphatic invasion in primary breast cancer. Ann Surg Oncol.

[3] Hou G, Zhang S, Zhang X, Wang P, Hao X, Zhang J (2013). Clinical pathological characteristics and prognostic analysis of 1,013 breast cancer patients with diabetes. Breast Cancer Res Treat.

[4] Liao S, Li J, Wang L, Zhang Y, Wang C, Hu M, Ma B, Wang G, Sun S (2010). Type 2 diabetes mellitus and characteristics of breast cancer in China. Asian Pacific journal of cancer prevention : APJCP.

[5] Luo J, Virnig B, Hendryx M, Wen S, Chelebowski R, Chen C, Rohan T, Tinker L, Wactawski-Wende J, Lessin L (2014). Diabetes, diabetes treatment and breast cancer prognosis. Breast Cancer Res Treat.

[6] Srokowski TP, Fang S, Hortobagyi GN, Giordano SH (2009). Impact of diabetes mellitus on complications and outcomes of adjuvant chemotherapy in older patients with breast cancer. Journal of clinical oncology : official journal of the American Society of Clinical Oncology.

[7] Larsson SC, Mantzoros CS, Wolk A (2007). Diabetes mellitus and risk of breast cancer: a meta-analysis. International journal of cancer Journal international du cancer.

[8] Liao S, Li J, Wei W, Wang L, Zhang Y, Li J, Wang C, Sun S (2011). Association between diabetes mellitus and breast cancer risk: a meta-analysis of the literature. Asian Pacific journal of cancer prevention : APJCP.

[9] Peairs KS, Barone BB, Snyder CF, Yeh HC, Stein KB, Derr RL, Brancati FL, Wolff AC (2011). Diabetes mellitus and breast cancer outcomes: a systematic review and meta-analysis. Journal of clinical oncology : official journal of the American Society of Clinical Oncology.

[10] Xue F, Michels KB (2007). Diabetes, metabolic syndrome, and breast cancer: a review of the current evidence. Am J Clin Nutr.

[11] Rajpathak SN, He M, Sun Q, Kaplan RC, Muzumdar R, Rohan TE, Gunter MJ, Pollak M, Kim M, Pessin JE (2012). Insulin-like growth factor axis and risk of type 2 diabetes in women. Diabetes.

[12] Shimizu C, Hasegawa T, Tani Y, Takahashi F, Takeuchi M, Watanabe T, Ando M, Katsumata N, Fujiwara Y (2004). Expression of insulin-like growth factor 1 receptor in primary breast cancer: immunohistochemical analysis. Hum Pathol.

[13] Rose DP, Vona-Davis L (2012). The cellular and molecular mechanisms by which insulin influences breast cancer risk and progression. Endocr Relat Cancer.

[14] Internal Diabetes Federation: Diabetes atlas. In., 7th edition EDN; 2015.

[15] Zhang W, Liu HT: MAPK signal pathways in the regulation of cell proliferation in mammalian cells. (1001–0602 (Print)). 10.1038/sj.cr.7290105 11942415

[16] Belfiore A, Malaguarnera R (2011). Insulin receptor and cancer. Endocr Relat Cancer.

[17] Werner H, Chantelau EA. Differences in bioactivity between human insulin and insulin analogues approved for therapeutic use- compilation of reports from the past 20 years. Diabetology & Metabolic Syndrome. 2011;3 10.1186/1758-5996-3-13 PMC3160352 21714872

[18] Slieker LJ, Brooke GS, DiMarchi RD, Flora DB, Green LK, Hoffmann JA, Long HB, Fan L, Shields JE, Sundell KL (1997). Modifications in the B10 and B26-30 regions of the B chain of human insulin alter affinity for the human IGF-I receptor more than for the insulin receptor. Diabetologia.

[19] Tao Y, Pinzi V, Bourhis J, Deutsch E (2007). Mechanisms of disease: signaling of the insulin-like growth factor 1 receptor pathway--therapeutic perspectives in cancer. Nat Clin Pract Oncol.

[20] Bronsveld HK, ter Braak B, Karlstad O, Vestergaard P, Starup-Linde J, Bazelier MT, De Bruin ML, de Boer A, Siezen CL, van de Water B (2015). Treatment with insulin (analogues) and breast cancer risk in diabetics; a systematic review and meta-analysis of in vitro, animal and human evidence. Breast cancer research : BCR.

[21] ter Braak B, Siezen C, Speksnijder EN, Koedoot E, van Steeg H, Salvatori DC, van de Water B, van der Laan JW (2015). Mammary gland tumor promotion by chronic administration of IGF1 and the insulin analogue AspB10 in the p53R270H/(+)WAPCre mouse model. Breast cancer research : BCR.

[22] Lanzino M, Morelli C Fau - Garofalo C, Garofalo C Fau - Panno ML, Panno Ml Fau - Mauro L, Mauro L Fau - Ando S, Ando S Fau - Sisci D, Sisci D: Interaction between estrogen receptor alpha and insulin/IGF signaling in breast cancer. (1873–5576 (Electronic)).

[23] Moller S, Jensen MB, Ejlertsen B, Bjerre KD, Larsen M, Hansen HB, Christiansen P, Mouridsen HT (2008). The clinical database and the treatment guidelines of the Danish breast cancer cooperative group (DBCG); its 30-years experience and future promise. Acta oncologica (Stockholm, Sweden).

[24] Bronsveld HK, Jensen V, Vahl P, De Bruin ML, Cornelissen S, Sanders J, Auvinen A, Haukka J, Andersen M, Vestergaard P (2017). Diabetes and breast cancer subtypes. PLoS One.

[25] Blows FM, Driver KE, Schmidt MK, Broeks A, van Leeuwen FE, Wesseling J, Cheang MC, Gelmon K, Blomqvist C, Nielsen TO (2010). Subtyping of breast cancer by immunohistochemistry to investigate a relationship between subtype and short and long term survival: a collaborative analysis of data for 10,159 cases from 12 studies. PLoS Med.

[26] Murphy LC, Niu Y, Snell L, Watson P (2004). Phospho-serine-118 estrogen receptor-alpha expression is associated with better disease outcome in women treated with tamoxifen. Clinical cancer research : an official journal of the American Association for Cancer Research.

[27] Beelen K, Opdam M, Severson TM, Koornstra RH, Vincent AD, Wesseling J, Muris JJ, Berns EM, Vermorken JB, van Diest PJ (2014). Phosphorylated p-70S6K predicts tamoxifen resistance in postmenopausal breast cancer patients randomized between adjuvant tamoxifen versus no systemic treatment. Breast cancer research : BCR.

[28] Baricevic I, Jones DR, Roberts DL, Lutzen A, Lundby A, Worm J, Hansen BF, Renehan AG (2015). A framework for the in vitro evaluation of cancer-relevant molecular characteristics and mitogenic potency of insulin analogues. Carcinogenesis.

[29] Hartog H, Horlings HM, van der Vegt B, Kreike B, Ajouaou A, van de Vijver MJ, Marike Boezen H, de Bock GH, van der Graaf WT, Wesseling J (2011). Divergent effects of insulin-like growth factor-1 receptor expression on prognosis of estrogen receptor positive versus triple negative invasive ductal breast carcinoma. Breast Cancer Res Treat.

[30] SAS Data Analysis, Zero-inflated Negative Binomial Regression [https://stats.idre.ucla.edu/r/dae/zinb/].

[31] Brand JS, Onland-Moret NC, Eijkemans MJ, Tjonneland A, Roswall N, Overvad K, Fagherazzi G, Clavel-Chapelon F, Dossus L, Lukanova A (2015). Diabetes and onset of natural menopause: results from the European prospective investigation into cancer and nutrition. Human reproduction (Oxford, England).

[32] van Buuren S, Groothuis-Oudshoorn K: mice: Multivariate Imputation by Chained Equations in R. 2011 2011, 45(3):67.

[33] Jalving M, Gietema JA, Lefrandt JD, de Jong S, Reyners AK, Gans RO, de Vries EG (2010). Metformin: taking away the candy for cancer?. European journal of cancer (Oxford, England : 1990).

[34] Nardon E, Buda I, Stanta G, Buratti E, Fonda M, Cattin L (2003). Insulin-like growth factor system gene expression in women with type 2 diabetes and breast cancer. J Clin Pathol.

[35] Xin C, Jing D, Jie T, Wu-Xia L, Meng Q, Ji-Yan L (2015). The expression difference of insulin-like growth factor 1 receptor in breast cancers with or without diabetes. J Cancer Res Ther.

[36] Pierre-Eugene C, Pagesy P, Nguyen TT, Neuille M, Tschank G, Tennagels N, Hampe C, Issad T (2012). Effect of insulin analogues on insulin/IGF1 hybrid receptors: increased activation by glargine but not by its metabolites M1 and M2. PLoS One.

[37] Shukla A, Grisouard J, Ehemann V, Hermani A, Enzmann H, Mayer D (2009). Analysis of signaling pathways related to cell proliferation stimulated by insulin analogs in human mammary epithelial cell lines. Endocr Relat Cancer.

[38] Teng JA, Hou RL, Li DL, Yang RP, Qin J (2011). Glargine promotes proliferation of breast adenocarcinoma cell line MCF-7 via AKT activation. Horm Metab Res.

[39] Ter Braak B, Siezen CL, Kannegieter N, Koedoot E, van de Water B, van der Laan JW (2014). Classifying the adverse mitogenic mode of action of insulin analogues using a novel mechanism-based genetically engineered human breast cancer cell panel. Arch Toxicol.

[40] Mengel M, von Wasielewski R, Wiese B, Rudiger T, Muller-Hermelink HK, Kreipe H (2002). Inter-laboratory and inter-observer reproducibility of immunohistochemical assessment of the Ki-67 labelling index in a large multi-centre trial. J Pathol.

[41] O'Leary TJ (2001). Standardization in immunohistochemistry. Applied immunohistochemistry & molecular morphology : AIMM.

[42] Magkou C, Nakopoulou L, Zoubouli C, Karali K, Theohari I, Bakarakos P, Giannopoulou I (2008). Expression of the epidermal growth factor receptor (EGFR) and the phosphorylated EGFR in invasive breast carcinomas. Breast cancer research : BCR.

[43] Yang Z, Ming XF: mTOR signalling: the molecular interface connecting metabolic stress, aging and cardiovascular diseases. (1467-789X (Electronic)). 10.1111/j.1467-789X.2012.01038.x 23107260

[44] Jia G, Aroor AR, Martinez-Lemus LA, Sowers JR: Overnutrition, mTOR signaling, and cardiovascular diseases. (1522–1490 (Electronic)). 10.1152/ajpregu.00262.2014 PMC4233289 25253086

[45] Novosyadlyy R, Lann DE, Vijayakumar A, Rowzee A, Lazzarino DA, Fierz Y, Carboni JM, Gottardis MM, Pennisi PA, Molinolo AA (2010). Insulin-mediated acceleration of breast cancer development and progression in a nonobese model of type 2 diabetes. Cancer Res.

[46] Cusi K, Maezono K, Osman A, Pendergrass M, Patti ME, Pratipanawatr T, DeFronzo RA, Kahn CR, Mandarino LJ (2000). Insulin resistance differentially affects the PI 3-kinase- and MAP kinase-mediated signaling in human muscle. J Clin Invest.

[47] Jiang ZY, Lin YW, Clemont A, Feener EP, Hein KD, Igarashi M, Yamauchi T, White MF, King GL (1999). Characterization of selective resistance to insulin signaling in the vasculature of obese Zucker (fa/fa) rats. J Clin Invest.

[48] Di Cosimo S, Sathyanarayanan S, Bendell JC, Cervantes A, Stein MN, Brana I, Roda D, Haines BB, Zhang T, Winter CG (2015). Combination of the mTOR inhibitor ridaforolimus and the anti-IGF1R monoclonal antibody dalotuzumab: preclinical characterization and phase I clinical trial. Clinical cancer research : an official journal of the American Association for Cancer Research.

[49] Ma CX, Suman VJ, Goetz M, Haluska P, Moynihan T, Nanda R, Olopade O, Pluard T, Guo Z, Chen HX (2013). A phase I trial of the IGF-1R antibody Cixutumumab in combination with temsirolimus in patients with metastatic breast cancer. Breast Cancer Res Treat.

[50] Gallagher EJ, Fierz Y, Vijayakumar A, Haddad N, Yakar S, LeRoith D (2012). Inhibiting PI3K reduces mammary tumor growth and induces hyperglycemia in a mouse model of insulin resistance and hyperinsulinemia. Oncogene.

[51] Li R, Pourpak A Fau - Morris SW, Morris SW: Inhibition of the insulin-like growth factor-1 receptor (IGF1R) tyrosine kinase as a novel cancer therapy approach. (1520–4804 (Electronic)). 10.1021/jm9002395 PMC2888655 19610618

