# Supplementary material for: The association of diabetes mellitus and insulin treatment with expression of insulin-related proteins in breast tumors
Source: BMC Cancer. 2018 Feb 27;18:224. doi: 10.1186/s12885-018-4072-8 (PMC6389252; doi:10.1186/s12885-018-4072-8)
Supplement: Supplementary file 1 — Antibodies used for immunohistochemical assays. Table S2 Overview of the number of positively stained and unevaluable immunohistochemical markers; with for the evaluable cores the median percent of tumor cells with moderate to strong protein expression. Table S3. Average Body Mass Index of breast cancer patients in subgroups of menopausal status, in the ten imputed datasets (% (n)). Table S4. Characteristics of breast cancer patients with and without diabetes and of insulin and non-insulin users. Table S5. Patient characteristics and medication use among women with type 1 and type 2 diabetes. Table S6. Numbers and proportions of tumor protein expression status of women with diabetes, type 2 diabetes and without diabetes in all women and in subgroups of menopausal status. Table S7. Odds ratios for tumor protein expression status of women with type 2 diabetes compared to women without diabetes, and of women with and without diabetes in subgroups of menopausal status. Table S8. Number and proportion of tumor protein expression status of women with type 1 and type 2 diabetes treated with insulin (human and analogues) and without insulin. Table S9. Number and proportion of tumor protein expression status of women with diabetes treated with insulin and without insulin in subgroups of menopausal status and ER tumor status. Table S10. Odds ratios for tumor protein expression status of women with diabetes; treated with insulin analogues compared to women treated with human insulin; type 1 compared to type 2 insulin users; treated with insulin compared to women not treated with insulin in subgroups of menopausal status and in subgroups of ER tumor status. (DOCX 88 kb) [file 12885_2018_4072_MOESM1_ESM.docx]

**Additional files**

**Table S1** Antibodies used for immunohistochemical assays.

| **Protein name** | **Successful staining** | **Epitope** | **Clone** | **Manufacturer** | **Catalogue number** | **Dilution** | **Localization** |
| --- | --- | --- | --- | --- | --- | --- | --- |
| p-ER | v | Ser118 | 16J4 | Cell Signaling | 2511 | 1/1200 | Nuclear |
| EGFR | v |  | 5B7 | Roche | 790-4347 | Ready to use dispenser | Membrane |
| p-ERK1/2 (p-p44/42 MAPK) | v | Thr202/Tyr204 | D13.14.4E | Cell Signaling | 4370 | 1/400 | Nuclear/Cytoplasmic |
| p-mTOR | v | Ser2448 | 49F9 | Cell Signaling | 2976 | 1/200 | Cytoplasmic |
| p-P70S6K* |  | Thr389 | 1A5 | Cell Signaling | 9206 | 1/300 |  |
| p-Akt* |  | Thr308 | C31E5E | Cell Signaling | 2965 | 1/50 |  |
| p-Akt* |  | Ser473 | D9E | Cell Signaling | 4060 | 1/25 | Cytoplasmic/Nuclear |
| IGF1R | v |  | G11 | Roche | 790-4346 | Ready to use dispenser | Membrane/Cytoplasmic |
| INSR* |  |  | CT-3 | Merck Millipore | 05-1104 | <1/50 | Membrane |

* antibody did not show sufficient validity and reliability on human breast tissue**.** P-Akt (Th 308) and p-P70-S6K (Thr389) showed variations in staining pattern, both antibodies appeared *not* to be validated for IHC-P by Cell Signaling. The p-Akt (Ser473) antibody was validated for IHC-P, but showed minimal positive signal on mamma tissue. The previously validated INSR antibody (MAB1139, Calbiochem) appeared not te be available anymore; therefore in data sheet recommended replacement was used. However, INSR staining with this replacement showed weak to no signal in tumor tissue although endothelial cells showed moderate to strong positivity. Using less diluted antibody and/or a manual staining procedure did not lead to increased signal. *p-ER= phosphorylated estrogen receptor, EGFR=epidermal growth factor receptor, p-ERK= phosphorylated extracellular signal-regulated kinase, p-mTOR=phosphorylated mechanistic target of rapamycin, IGF1R=insulin growth factor 1 receptor, INSR=insulin receptor, p-AKT=phosphorylated protein kinase B, p-P70S6K=phosphorylated ribosomal protein S6 kinase.*

**Table S2** Overview of the number of positively stained and unevaluable immunohistochemical markers; with for the evaluable cores the median percent of tumor cells with moderate to strong protein expression.

| **Tumor IHC markers n=312** | **Intensity n (%)** | | **Classification based on intensity and percentage positive cells,**  **n (% of the evaluable cores)** | | **Median (IQ range)** |
| --- | --- | --- | --- | --- | --- |
| p-ER | Medium/strong intensity | 237 (76.0) | Positive | 136 (47.1) | 0 (0, 40) |
|  |  |  | Negative | 101 (34.9) |  |
|  | Weak intensity | 52 (16.7) | Negative | 52 (18.0) |  |
|  | Unevaluable | 23 (7.4) |  |  |  |
| EGFR | Medium/strong intensity | 263 (84.3) | Positive | 27 (9.0) | 0 (0, 0) |
|  |  |  | Negative | 236 (78.9) |  |
|  | Weak intensity | 36 (11.5) | Negative | 36 (12.0) |  |
|  | Unevaluable | 13 (4.2) |  |  |  |
| p-ERK1/2 | Medium/strong intensity | 279 (89.4) | Positive | 163 (54.7) | 10 (0, 40) |
|  |  |  | Negative | 116 (38.9) |  |
|  | Weak intensity | 19 (6.1) | Negative | 19 (6.4) |  |
|  | Unevaluable | 14 (4.5) |  |  |  |
| p-mTOR | Medium/strong intensity | 266 (85.3) | Positive | 170 (58.6) | 40 (0, 75) |
|  |  |  | Negative | 96 (33.1) |  |
|  | Weak intensity | 24 (7.7) | Negative | 24 (8.3) |  |
|  | Unevaluable | 22 (7.1) |  |  |  |
| IGF1R* | Medium/strong intensity | 225 (72.1) | Positive | 214 (72.8) |  |
|  |  |  | Negative | 11 (3.7) |  |
|  | Weak intensity | 69 (22.1) | Negative | 69 (23.5) |  |
|  | Unevaluable | 18 (5.8) |  |  |  |

- Median percent of tumor cells for IGF1R are not presented since we did not continuously score the percentages of tumors cells with moderate to strong staining. IHC for some of the tumors was not evaluable because the tumor tissue core did not include (invasive) breast tumor tissue or the core was missing. *p-ER= phosphorylated estrogen receptor, EGFR=epidermal growth =factor receptor, p-ERK= phosphorylated extracellular signal-regulated kinase, p-mTOR=phosphorylated mechanistic target of rapamycin, IGF1R=insulin growth factor 1 receptor.*

**Table S3** Average Body Mass Index of breast cancer patients in subgroups of menopausal status, in the ten imputed datasets (% (n)).

|  | **Premenopausal women with breast cancer** | | |
| --- | --- | --- | --- |
|  | **Diabetes** (n=110^x^10) |  | **No Diabetes** (n=49^x^10) |
| **BMI in kg/m^2 a^** |  |  |  |
| <25 (normal) | 33.0 (363) |  | 48.6 (238) |
| ≥25 (overweight) | 27.3 (300) |  | 43.9 (215) |
| ≥30 (obese) | 39.7 (437) |  | 7.6 (37) |
|  |  | | |
|  | **Postmenopausal women with breast cancer** | | |
|  | **Diabetes** (N=101^x^10) |  | **No Diabetes** (N=52^x^10) |
| **BMI in kg/m^2 a^** |  |  |  |
| <25 (normal) | 26.1 (264) |  | 49.2 (256) |
| ≥25 (overweight) | 36.7 (371) |  | 32.1 (167) |
| ≥30 (obese) | 37.1 (375) |  | 18.7 (97) |

^a^ Closest measure prior to breast cancer diagnosis. Imputation was done separately for pre- and postmenopausal women. *BMI=Body Mass Index*

**Table S4** Characteristics of breast cancer patients with and without diabetes and of insulin and non-insulin users.

|  |  | **Women with breast cancer** | | | | | | |
| --- | --- | --- | --- | --- | --- | --- | --- | --- |
|  |  | **Diabetes**  (n=211) |  | **No Diabetes** (n=101) |  | **Insulin** ^§^  (n=53) |  | **No Insulin** ^†^  (n=158) |
| **Age**, median (IQ range) ^a, b^ |  |  |  |  |  |  |  |  |
| ≤ 50 years |  | 47 (43-50) |  | 47.0 (43-50) |  | 47 (43-49) |  | 48 (44-50) |
| > 50 years |  | 67 (60-75) |  | 67.0 (62-73) |  | 65 (58-69) |  | 68 (61-75) |
| **BMI in kg/m^2^**, median (IQ range) ^c^ |  |  |  |  |  |  |  |  |
| Premenopausal women |  | 28.3 (23.9-33.5) | ^≠^ | 25.2 (22.1-26.6) |  | 24.3 (22.3-28.9) | ^≠^ | 30.7 (25.9-34.3) |
| Postmenopausal women |  | 29.0 (24.6-32.0) | ^≠^ | 24.7 (21.0-27.0) |  | 28.3 (23.2-35.0) |  | 29.1 (25.2-31.2) |
|  |  |  |  |  |  |  |  |  |
|  |  | **% (n)** |  | **% (n)** |  | **% (n)** |  | **% (n)** |
| **Year of breast cancer diagnoses** ^a^ |  |  |  |  |  |  |  |  |
| 2000-2002 |  | 12.8 (27) |  | 6.9 (7) |  | 20.7 (11) |  | 10.1 (16) |
| 2003-2004 |  | 15.6 (33) |  | 16.8 (17) |  | 13.2 (7) |  | 16.5 (26) |
| 2005-2006 |  | 17.5 (37) |  | 33.7 (34) |  | 17.0 (9) |  | 17.7 (28) |
| 2007-2008 |  | 27.5 (58) |  | 18.8 (19) |  | 28.3 (15) |  | 27.2 (43) |
| 2009-2010 |  | 26.6 (56) |  | 23.8 (24) |  | 20.8 (11) |  | 28.5 (45) |
| **Menopausal status** ^b^ |  |  |  |  |  |  |  |  |
| Pre |  | 52.1 (110) |  | 48.5 (49) |  | 64.2 (34) | ^≠^ | 48.1 (76) |
| Post |  | 47.9 (101) |  | 51.5 (52) |  | 35.9 (19) | ^≠^ | 51.9 (82) |
|  |  |  |  |  |  |  |  |  |
| **Morphology** |  |  |  |  |  |  |  |  |
| Ductal |  | 75.8 (160) |  | 70.3 (71) |  | 73.6 (39) |  | 76.6 (121) |
| Lobular |  | 7.6 (16) |  | 10.9 (11) |  | 11.3 (6) |  | 6.3 (10) |
| Other |  | 16.6 (35) |  | 18.8 (19) |  | 15.1 (8) |  | 17.1 (27) |
| **Tumour size in mm** |  |  |  |  |  |  |  |  |
| ≤ 20 |  | 57.8 (122) |  | 57.4 (58) |  | 62.3 (33) |  | 56.3 (89) |
| 21-50 |  | 36.5 (77) |  | 39.6 (40) |  | 32.1 (17) |  | 38.0 (60) |
| >50 |  | 5.7 (12) |  | <5 (<5) ^ǂ^ |  | <6 (<5) ^ǂ^ |  | 5.7 (9) |
| **Number of positive lymph nodes** |  |  |  |  |  |  |  |  |
| 0 |  | 50.3 (102) |  | 54.0 (54) |  | 47.1 (24) |  | 51.3 (78) |
| 1-3 |  | 32.5 (66) |  | 26.0 (26) |  | 35.3 (18) |  | 31.6 (48) |
| >3 |  | 17.2 (35) |  | 20.0 (20) |  | 17.7 (9) |  | 17.1 (26) |
| **Grade** |  |  |  |  |  |  |  |  |
| Grade 1 |  | 20.3 (41) |  | 19.0 (19) |  | 26.0 (13) |  | 18.4 (28) |
| Grade 2 |  | 35.6 (72) | ^≠^ | 51.0 (51) |  | 38.0 (19) |  | 34.9 (53) |
| Grade 3 |  | 44.1 (89) |  | 30.0 (30) |  | 36.0 (18) |  | 46.7 (71) |
| **ER** |  |  |  |  |  |  |  |  |
| Positive |  | 77.6 (163) |  | 86.1 (87) |  | 81.1 (43) |  | 76.4 (120) |
| Negative |  | 22.4 (47) |  | 13.9 (14) |  | 18.9 (10) |  | 23.6 (37) |
| **PR** |  |  |  |  |  |  |  |  |
| Positive |  | 64.4 (136) |  | 72.3 (73) |  | 71.7 (38) |  | 62.0 (98) |
| Negative |  | 35.6 (75) |  | 27.7 (28) |  | 28.3 (15) |  | 38.0 (60) |
| **HER2** |  |  |  |  |  |  |  |  |
| Positive |  | 10.5 (22) |  | 17.8 (18) |  | <7 (<5) ^ǂ^ |  | 11.4 (18) |
| Negative |  | 89.5 (187) |  | 82.2 (83) |  | 92.2 (47) |  | 88.6 (140) |

^a^ Matching variable, ^b^ At breast cancer diagnosis, ^c^ Closest measure prior to breast cancer diagnosis, ^d^ Chi-square test. Missing values are not shown, therefore the sum of the categories does not add up to the total number of patients for grade, ER and HER2. ^§^ Women with diabetes treated with insulin (analogues) regardless the use of concomitant noninsulin antidiabetic drugs. ^†^ Women with diabetes treated only with diet and exercise and users of noninsulin antidiabetic drugs only. ^≠^ statistically significant p <0.05.^ǂ^ Exact numbers <5 with percentages cannot be shown according to regulations of Statistics Denmark. *IQ=interquartile range, BMI=Body Mass Index, ER= Estrogen Receptor, PR=Progesterone Receptor, HER2=Human Epidermal growth factor Receptor 2.*

**Table S5** Patient characteristics and medication use among women with type 1 and type 2 diabetes ^*.^

|  | **Women with breast cancer and diabetes** | | | | |
| --- | --- | --- | --- | --- | --- |
|  | **Diabetes** ^§^  (n=211) |  | **Type 1 Diabetes** (n=25) |  | **Type 2 Diabetes** (n=186) |
| **Diabetes Type**, % (n) |  |  |  |  |  |
| Type 1 | 11.8 (25) |  |  |  |  |
| Type 2 | 88.2 (186) |  |  |  |  |
| **Age diabetes diagnosis**, median (IQ range) | 46.0 (34.0-58.0) |  | 23.0 (20.0-28.0) |  | 47.5 (38.0-61.0) |
| premenopausal | 36.0 (30.0-45.0) |  | 22.0 (20.0-27.0) |  | 39.0 (32.5-45.0) |
| postmenopausal | 59.0 (52.0-69.0) |  | 29.0 (20.0-47.0) |  | 61.0 (54.0-69.0) |
| **Menopausal status**, % (n) ^a^ |  |  |  |  |  |
| Pre | 51.9 (110) |  | 76.0 (19) |  | 48.9 (91) |
| Post | 48.1 (101) |  | 24.0 (6) |  | 51.1 (95) |
| **BMI in kg/m^2^,** % (n) ^b^ |  |  |  |  |  |
| <25 (normal) | 20.4 (43) |  | 40.0 (10) |  | 17.7 (33) |
| ≥25 (overweight) | 23.2 (49) |  | 24.0 (6) |  | 23.1 (43) |
| ≥ 30 (obese) | 32.2 (68) |  | <5 (<5) ^ǂ^ |  | 36.0 (67) |
| Missing | 24.2 (51) |  | 32.0 (8) |  | 23.1 (43) |
| **Diabetes duration in years**, mean ± SD | 8.9 ± 7.7 |  | 22.3 ± 7.3 |  | 7.1 ± 5.8 |
| **Diabetes treatment**, % (n) ^c^ |  |  |  |  |  |
| Diet and exercise | 39.8 (84) |  | - |  | 45.2 (84) |
| Non-insulin antidiabetic drugs only | 35.1 (74) |  | - |  | 39.8 (74) |
| Insulin only | 16.6 (35) |  | 100.0 (25) |  | 5.4 (10) |
| Insulin and non-insulin antidiabetic drugs | 8.5 (18) |  | - |  | 9.7 (18) |
| **Exposure time** **in years,** mean ± SD ^d^ |  |  |  |  |  |
| Any antidiabetic drugs | 6.8 ± 4.1 |  | 10.1 ± 3.5 |  | 6.0 ± 3.8 |
| Insulin | 8.4 ± 4.2 |  | 10.1 ± 3.5 |  | 6.9 ± 4.2 |
| Non-insulin antidiabetic drugs | 5.5 ± 3.6 |  | - |  | 5.5 ± 3.6 |
| **Insulin type**, % (n) |  |  |  |  |  |
| Human insulin only | 13.3 (28) |  | 56.0 (14) |  | 7.5 (14) |
| Insulin analogues only | 1.4 (3) |  | - |  | 1.6 (3) |
| Human insulin and insulin analogues | 10.4 (22) |  | 44.0 (11) |  | 5.9 (11) |
| **Metformin**, % (n) |  |  |  |  |  |
| Among non-insulin antidiabetic drug only users | 24.2 (51) |  | - |  | 27.4 (51) |
| Among insulin and non-insulin antidiabetic drug users | 5.7 (12) |  | - |  | 6.5 (12) |

^*^ Used for imputation. ^a^ At breast cancer diagnosis, ^b^ Closest measure prior to breast cancer diagnosis ^c^ at least 2 prescriptions of an antidiabetic drug were prescribed in the period up to one year prior to breast cancer diagnosis, ^d^ defined as time from age of start of the antidiabetic drug till age of breast cancer diagnosis. ^§^ All women with diabetes. ^ǂ^ exact numbers <5 with percentages cannot be shown according to regulations of Statistics Denmark. *IQ=interquartile range, SD=standard deviation, BMI=Body Mass Index.*

**Table S6** Numbers and proportions of tumor protein expression status, by immunohistochemical markers, of women with diabetes, type 2 diabetes and without diabetes in all women and in subgroups of menopausal status.

|  | **Women with breast cancer** | | | | |  | **Premenopausal women**  **with breast cancer** | | |  | **Postmenopausal women**  **with breast cancer** | | |
| --- | --- | --- | --- | --- | --- | --- | --- | --- | --- | --- | --- | --- | --- |
|  | **Diabetes** ^§^  (n=211) |  | **Type 2 Diabetes** ^†^ (n=186) |  | **No Diabetes**  (n=101) |  | **Diabetes**  (n=110) |  | **No Diabetes**  (n=49) |  | **Diabetes**  (n=101) |  | **No Diabetes**  (n=52) |
| **Tumor IHC marker** | **% (n)** |  | **% (n)** |  | **% (n)** |  | **% (n)** |  | **% (n)** |  | **% (n)** |  | **% (n)** |
| p-ER - | 54.4 (105) |  | 52.6 (90) |  | 50.0 (48) |  | 63.1 (65) |  | 54.4 (25) |  | 44.4 (40) |  | 46.0 (23) |
| P-ER + | 45.6 (88) |  | 47.4 (81) |  | 50.0 (48) |  | 36.9 (38) |  | 45.7 (21) |  | 55.6 (50) |  | 54.0 (27) |
| EGFR - | 90.0 (181) |  | 90.4 (161) |  | 92.9 (91) |  | 90.3 (93) |  | 93.8 (45) |  | 89.8 (88) |  | 92.0 (46) |
| EGFR + | 10.0 (20) |  | 9.6 (17) |  | 7.1 (7) |  | 9.7 (10) |  | <7 (<5) ^ǂ^ |  | 10.2 (10) |  | <9 (<5) ^ǂ^ |
| p-ERK 1/2 - | 46.7 (93) |  | 46.6 (82) |  | 42.4 (42) |  | 41.8 (43) |  | 44.9 (22) |  | 52.1 (50) |  | 40.0 (20) |
| p-ERK 1/2 + | 53.3 (106) |  | 53.4 (94) |  | 57.6 (57) |  | 58.3 (60) |  | 55.1 (27) |  | 47.9 (46) |  | 60.0 (30) |
| p-mTOR - | 43.1 (84) |  | 44.5 (77) |  | 37.9 (36) |  | 43.7 (45) |  | 35.6 (16) |  | 42.4 (39) |  | 40.0 (20) |
| p-mTOR + | 56.9 (111) |  | 55.5 (96) |  | 62.1 (59) |  | 56.3 (58) |  | 64.4 (29) |  | 57.6 (53) |  | 60.0 (30) |
| IGF1R - | 27.9 (55) |  | 30.1 (52) |  | 25.8 (25) |  | 26.7 (28) |  | 29.8 (14) |  | 29.4 (27) |  | 22.0 (11) |
| IGF1R + | 72.1 (142) |  | 69.9 (121) |  | 74.2 (72) |  | 73.3 (77) |  | 70.2 (33) |  | 70.7 (65) |  | 78.0 (39) |

The sum of the categories for the tumor markers does not add up to the total number of patients due to unevaluable stainings for some patients*^.^* ^§^ All women with diabetes, type 1 and type 2. † Women with type 2 diabetes only. ^ǂ^ Exact numbers <5 with percentages cannot be shown according to regulations of Statistics Denmark. *p-ER= phosphorylated estrogen receptor, EGFR=epidermal growth =factor receptor, p-ERK= phosphorylated extracellular signal-regulated kinase, p-mTOR=phosphorylated mechanistic target of rapamycin, IGF1R=insulin growth factor 1 receptor.*

**Table S7** Crude and logistic odds ratios for tumor protein expression status, by immunohistochemical markers, of women with type 2 diabetes compared to women without diabetes, and of women with and without diabetes in subgroups of menopausal status, using logistic regression.

|  | **Independent variable of exposure** | | | | | | |
| --- | --- | --- | --- | --- | --- | --- | --- |
| **Dependent variable ^*^** | **crude OR (95% CI)** | **P** | |  | **adjusted OR ^#^ (95% CI)** | | **P** |
| **Women with breast cancer** |  | | |  |  | | |
|  | **Type 2 Diabetes** ^§^ **vs. No Diabetes** | | |  | **Type 2 Diabetes** ^§^  **vs. No Diabetes** | | |
| p-ER + | 0.90 (0.55-1.48) | | 0.68 |  | 1.12 (0.65-1.92) | 0.69 | |
| EGFR + | 1.37 (0.55-3.43) | | 0.50 |  | 1.64 (0.63-4.28) | 0.31 | |
| p-ERK 1/2 + | 0.85 (0.51-1.39) | | 0.51 |  | 0.88 (0.52-1.48) | 0.62 | |
| p-mTOR + | 0.76 (0.46-1.27) | | 0.29 |  | 0.85 (0.50-1.46) | 0.56 | |
| IGF1R + | 0.81 (0.46-1.41) | | 0.45 |  | 0.83 (0.46-1.49) | 0.54 | |
| **Premenopausal women with**  **breast cancer** | | |  |  |  |  | |
|  | **Diabetes vs. No Diabetes** | |  |  | **Diabetes vs. No Diabetes** |  | |
| p-ER + | 0.70 (0.34-1.41) | | 0.31 |  | 0.88 (0.42-1.88) | 0.75 | |
| EGFR + | 1.61 (0.42-6.15) | | 0.48 |  | 2.03 (0.52-7.99) | 0.31 | |
| p-ERK 1/2 + | 1.14 (0.57-2.26) | | 0.71 |  | 1.19 (0.58-2.44) | 0.63 | |
| p-mTOR + | 0.71 (0.35-1.47) | | 0.36 |  | 0.82 (0.39-1.75) | 0.61 | |
| IGF1R + | 1.17 (0.55-2.50) | | 0.69 |  | 1.25 (0.57-2.77) | 0.58 | |
| **Postmenopausal women with**  **breast cancer** | | | |  |  |  | |
|  | **Diabetes vs. No Diabetes** | |  |  | **Diabetes vs. No Diabetes** |  | |
| p-ER + | 1.07 (0.53-2.13) | | 0.86 |  | 1.21 (0.58-2.51) | 0.61 | |
| EGFR + | 1.31 (0.39-4.40) | | 0.67 |  | 1.59 (0.45-5.63) | 0.48 | |
| p-ERK 1/2 + | 0.61 (0.31-1.23) | | 0.17 |  | 0.62 (0.31-1.26) | 0.19 | |
| p-mTOR + | 0.91 (0.45-1.83) | | 0.78 |  | 0.98 (0.47-2.00) | 0.95 | |
| IGF1R + | 0.69 (0.30-1.52) | | 0.35 |  | 0.67 (0.29-1.52) | 0.34 | |

^*^ Logistic regression for tumor IHC marker as the dependent variable, with a negative staining of the tumor marker as reference category. ^§^ Women with type 2 diabetes only. ^#^ Adjusted for menopause (pre/post) at breast cancer diagnosis and BMI closest measure prior to breast cancer diagnosis (continuous). Women with diabetes were matched on age at breast cancer diagnosis to women without diabetes. *p-ER= phosphorylated estrogen receptor, EGFR=epidermal growth =factor receptor, p-ERK= phosphorylated extracellular signal-regulated kinase, p-mTOR=phosphorylated mechanistic target of rapamycin, IGF1R=insulin growth factor 1 receptor, OR=Odds Ratio, CI=Confidence Interval.*

**Table S8** Number and proportion of tumor protein expression status, by immunohistochemical markers, of women with type 1 and type 2 diabetes treated with insulin (human and analogues) and without insulin.

|  | **Women with breast cancer and diabetes** | | | | | | | | | | |
| --- | --- | --- | --- | --- | --- | --- | --- | --- | --- | --- | --- |
|  | **Diabetes**  **with Insulin**^*^  (n=53) |  | **Diabetes with insulin analogues ^#^** (n=25) |  | **Diabetes**  **with human insulin**^§^ (n=28) |  | **Type 1 Diabetes with Insulin** ^*^ (n=25) |  | **Type 2 Diabetes with insulin** ^*^ (n=28) |  | **Diabetes**  **without insulin**^†^  (n=158) |
| **Tumor IHC marker** | **% (n)** |  | **% (n)** |  | **% (n)** |  | **% (n)** |  | **% (n)** |  | **% (n)** |
| p-ER - | 52.2 (24) |  | 68.2 (15) |  | 37.5 (9) |  | 68.2 (15) |  | 37.5 (9) |  | 55.1 (81) |
| P-ER + | 47.8 (22) |  | 31.8 (7) |  | 62.5 (15) |  | 31.8 (7) |  | 62.5 (15) |  | 44.9 (66) |
| EGFR - | 85.4 (41) |  | 90.9 (20) |  | 80.8 (21) |  | 87.0 (20) |  | 84.0 (21) |  | 91.5 (140) |
| EGFR + | 14.6 (7) |  | <10 (<5) ^ǂ^ |  | 19.2 (5) |  | <14 (<5) ^ǂ^ |  | <17 (<5) ^ǂ^ |  | 8.5 (13) |
| p-ERK 1/2 - | 41.7 (20) |  | 34.8 (8) |  | 48.0 (12) |  | 47.8 (11) |  | 36.0 (9) |  | 48.3 (73) |
| p-ERK 1/2 + | 58.3 (28) |  | 65.2 (15) |  | 52.0 (13) |  | 52.2 (12) |  | 64.0 (16) |  | 52.7 (78) |
| p-mTOR - | 27.7 (13) |  | 27.3 (6) |  | 28.0 (7) |  | 31.8 (7) |  | 24.0 (6) |  | 48.0 (71) |
| p-mTOR + | 72.3 (34) |  | 72.7 (16) |  | 72.0 (18) |  | 68.2 (15) |  | 76.0 (19) |  | 52.0 (77) |
| IGF1R - | 16.0 (8) |  | <9 (<5) ^ǂ^ |  | 22.2 (6) |  | <13 (<5) ^ǂ^ |  | 19.2 (5) |  | 32.0 (47) |
| IGF1R + | 84.0 (42) |  | 91.3 (21) |  | 77.8 (21) |  | 87.5 (21) |  | 80.8 (21) |  | 68.0 (100) |

The sum of the categories for the tumor markers does not add up to the total number of patients due to unevaluable stainings for some patients*.* ^*^ Women with diabetes treated with insulin (analogues) regardless the use of concomitant non-insulin antidiabetic drugs. ^#^ Women with diabetes treated with insulin analogues regardless the use of concomitant human insulin (n=22) or noninsulin antidiabetic drugs. ^§^ Women with diabetes treated with human insulin only regardless the use of concomitant noninsulin antidiabetic drugs. ^†^ Women with diabetes treated only with diet and exercise and users of non-insulin antidiabetic drugs only. ^ǂ^ Exact numbers <5 with percentages cannot be shown according to regulations of Statistics Denmark. *p-ER= phosphorylated estrogen receptor, EGFR=epidermal growth =factor receptor, p-ERK= phosphorylated extracellular signal-regulated kinase, p-mTOR=phosphorylated mechanistic target of rapamycin, IGF1R=insulin growth factor 1 receptor*

**Table S9** Number and proportion of tumor protein expression status, by immunohistochemical markers, of women with diabetes treated with insulin and without insulin in subgroups of menopausal status and ER tumor status.

|  | **Women with breast cancer and diabetes** | | | | | | | | | | | | | | |
| --- | --- | --- | --- | --- | --- | --- | --- | --- | --- | --- | --- | --- | --- | --- | --- |
|  | **Premenopausal** | | |  | **Postmenopausal** | | |  | **ER positive** | | |  | **ER negative** | | |
|  | **Diabetes with Insulin**^*^ (n=34) |  | **Diabetes without insulin**^†^ (n=76) |  | **Diabetes**  **With Insulin**^*^ (n=19) |  | **Diabetes without insulin**^†^  (n=82) |  | **Diabetes**  **with Insulin**^*^  (n=43) |  | **Diabetes without insulin**^†^ (n=121) |  | **Diabetes**  **with Insulin**^*^ (n=10) |  | **Diabetes without**  **Insulin**^†^  (n=38) |
| **Tumor IHC marker** | **% (n)** |  | **% (n)** |  | **% (n)** |  | **% (n)** |  | **% (n)** |  | **% (n)** |  | **% (n)** |  | **% (n)** |
| p-ER - | 63.3 (19) |  | 63.0 (46) |  | 31.3 (5) |  | 47.3 (35) |  | 43.2 (16) |  | 47.8 (53) |  | 88.9 (8) |  | 78.4 (29) |
| P-ER + | 36.7 (11) |  | 37.0 (27) |  | 68.8 (11) |  | 52.7 (39) |  | 56.8 (21) |  | 52.3 (58) |  | <12 (<5) ^ǂ^ |  | 21.6 (8) |
| EGFR - | 90.0 (27) |  | 90.4 (66) |  | 77.8 (14) |  | 92.5 (74) |  | 92.1 (35) |  | 99.1 (115) |  | 60.0 (6) |  | 68.4 (26) |
| EGFR + | <11 (<5) ^ǂ^ |  | 9.6 (7) |  | <23 (<5) ^ǂ^ |  | 7.5 (6) |  | <8 (<5) ^ǂ^ |  | <5 (<5) ^ǂ^ |  | <41 (<5) ^ǂ^ |  | 31.6 (12) |
| p-ERK 1/2 - | 43.3 (13) |  | 41.1 (30) |  | 38.9 (7) |  | 55.1 (43) |  | 42.1 (16) |  | 45.6 (52) |  | <41 (<5) ^ǂ^ |  | 57.9 (22) |
| p-ERK 1/2 + | 56.7 (17) |  | 58.9 (43) |  | 61.1 (11) |  | 44.9 (35) |  | 57.9 (22) |  | 54.4 (62) |  | 60.0 (6) |  | 42.1 (16) |
| p-mTOR - | 30.0 (9) |  | 49.3 (36) |  | <24 (<5) ^ǂ^ |  | 46.7 (35) |  | 18.9 (7) |  | 36.6 (41) |  | 60.0 (6) |  | 81.1 (30) |
| p-mTOR + | 70.0 (21) |  | 50.7 (37) |  | 76.5 (13) |  | 53.3 (40) |  | 81.1 (30) |  | 63.4 (71) |  | <41 (<5) ^ǂ^ |  | 18.9 (7) |
| IGF1R - | <10 (<5) ^ǂ^ |  | 34.3 (25) |  | 27.8 (5) |  | 29.7 (22) |  | <11 (<5) ^ǂ^ |  | 18.8 (21) |  | <41(<5) ^ǂ^ |  | 72.2 (26) |
| IGF1R + | 90.6 (29) |  | 65.8 (48) |  | 72.2 (13) |  | 70.3 (74) |  | 90.0 (36) |  | 81.3 (91) |  | 60.0 (6) |  | 27.8 (10) |

The sum of the categories for the tumor markers does not add up to the total number of patients due to unevaluable stainings for some patients. * Women with diabetes treated with insulin (analogues) regardless the use of concomitant non-insulin antidiabetic drugs. ^†^ Women with diabetes treated only with diet and exercise and users of non-insulin antidiabetic drugs only. ^ǂ^ Exact numbers <5 with percentages cannot be shown according to regulations of Statistics Denmark. *p-ER= phosphorylated estrogen receptor, EGFR=epidermal growth =factor receptor, p-ERK= phosphorylated extracellular signal-regulated kinase, p-mTOR=phosphorylated mechanistic target of rapamycin, IGF1R=insulin growth factor 1 receptor*

**Table S10** Crude and adjusted odds ratios for tumor protein expression status, by immunohistochemical markers, of women with diabetes; treated with insulin analogues compared to women treated with human insulin; type 1 compared to type 2 insulin users; treated with insulin compared to women not treated with insulin in subgroups of menopausal status and in subgroups of ER tumor status using logistic regression.

|  | **Independent variable of exposure** | | | | | | |
| --- | --- | --- | --- | --- | --- | --- | --- |
| **Dependent variable ^*^** | **crude OR (95% CI)** | **P** | |  | **adjusted OR ^#^ (95% CI)** | | **P** |
| **Women with breast cancer**  **and diabetes using insulin** | | | |  |  | | |
|  | **Insulin analogue**  ^ǂ^ **vs.**  **human Insulin** ^¥^ | | |  | **Insulin analogue** ^ǂ^  **vs.**  **human Insulin** ^¥^ | | |
| p-ER + | **0.28 (0.08-0.95)** | | **0.04** |  | 0.57 (0.22-1.49) | 0.25 | |
| EGFR + | 0.42 (0.07-2.42) | | 0.33 |  | 1.08 (0.23-5.13) | 0.93 | |
| p-ERK 1/2 + | 1.73 (0.54-5.54) | | 0.36 |  | 1.76 (0.70-4.38) | 0.23 | |
| p-mTOR + | 1.04 (0.29-3.74) | | 0.96 |  | 2.46 (0.91-6.63) | 0.08 | |
| IGF1R + | 3.00 (0.54-16.60) | | 0.21 |  | **4.94 (1.11-21.92)** | **0.04** | |
| **Women with breast cancer**  **and diabetes using insulin** | | |  |  |  |  | |
|  | **Type 1 Diabetes with Insulin** ^§^ **vs. Type 2 Diabetes with Insulin** ^§^ | | |  | **Type 1 Diabetes with Insulin** ^§^ **vs. Type 2 Diabetes with Insulin** ^§^ | | |
| p-ER + | **0.28 (0.08-0.95)** | | **0.04** |  | 0.27 (0.06-1.15) | 0.08 | |
| EGFR + | 0.79 (0.16-3.97) | | 0.77 |  | 1.04 (0.17-6.16) | 0.97 | |
| p-ERK 1/2 + | 0.61 (0.19-1.95) | | 0.41 |  | 0.69 (0.20-2.41) | 0.56 | |
| p-mTOR + | 0.68 (0.19-2.44) | | 0.55 |  | 0.76 (0.19-3.12) | 0.71 | |
| IGF1R + | 1.67 (0.35-1.88) | | 0.52 |  | 1.15 (0.21-6.22) | 0.87 | |
| **Premenopausal women with**  **breast cancer and diabetes** | | | |  |  |  | |
|  | **Insulin** ^§^ **vs. No Insulin** ^†^ | |  |  | **Insulin** ^§^ **vs. No Insulin** ^†^ |  | |
| p-ER + | 0.99 (0.41-2.38) | | 0.98 |  | 0.66 (0.25-1.74) | 0.40 | |
| EGFR + | 1.05 (0.25-4.36) | | 0.95 |  | 0.71 (0.16-3.23) | 0.66 | |
| p-ERK 1/2 + | 0.91 (0.39-2.16) | | 0.83 |  | 0.85 (0.34-2.09) | 0.72 | |
| p-mTOR + | 2.27 (0.92-5.61) | | 0.08 |  | 2.01 (0.78-5.17) | 0.15 | |
| IGF1R + | **5.04 (1.40-18.17)** | | **0.01** |  | **5.10 (1.36-19.14)** | **0.02** | |
| **Postmenopausal women with**  **breast cancer and diabetes** | | | |  |  |  | |
|  | **Insulin** ^§^ **vs. No Insulin** ^†^ | |  |  | **Insulin** ^§^ **vs. No Insulin** ^†^ |  | |
| p-ER + | 1.97 (0.62-6.24) | | 0.25 |  | 1.88 (0.58-6.09) | 0.29 | |
| EGFR + | 3.53 (0.88-14.12) | | 0.07 |  | 3.29 (0.80-13.56) | 0.10 | |
| p-ERK 1/2 + | 1.93 (0.68-5.50) | | 0.22 |  | 2.00 (0.69-5.79) | 0.20 | |
| p-mTOR + | 2.84 (0.85-9.53) | | 0.09 |  | 2.80 (0.83-9.47) | 0.10 | |
| IGF1R + | 1.10 (0.35-3.46) | | 0.87 |  | 1.08 (0.34-3.41) | 0.90 | |
| **Women with ER-positive**  **breast cancer and diabetes** | | |  |  |  |  | |
|  | **Insulin** ^§^ **vs. No Insulin** ^†^ | |  |  | **Insulin** ^§^ **vs. No Insulin** ^†^ |  | |
| p-ER + | 1.20 (0.57-2.54) | | 0.63 |  | 1.17 (0.52-2.64) | 0.70 | |
| EGFR + | 9.86 (1.00-97.80) | | 0.05 |  | 8.00 (0.73-87.51) | 0.09 | |
| p-ERK 1/2 + | 1.15 (0.55-2.42) | | 0.71 |  | 1.15 (0.53-2.48) | 0.72 | |
| p-mTOR + | 2.48 (1.00-6.14) | | 0.05 |  | 2.37 (0.93-6.03) | 0.07 | |
| IGF1R + | 2.08 (0.66-6.47) | | 0.21 |  | 1.97 (0.61-6.32) | 0.26 | |
| **Women with ER-negative**  **breast cancer and diabetes** | | |  |  |  |  | |
|  | **Insulin** ^§^ **vs. No Insulin** ^†^ | |  |  |  |  | |
| p-ER + | 0.45 (0.05-4.18) | | 0.48 |  |  |  | |
| EGFR + | 1.44 (0.34-6.09) | | 0.62 |  |  |  | |
| p-ERK 1/2 + | 2.06 (0.50-8.53) | | 0.32 |  |  |  | |
| p-mTOR + | 2.86 (0.63-12.92) | | 0.17 |  |  |  | |
| IGF1R + | 3.90 (0.91-16.80) | | 0.07 |  |  |  | |

^*^ Logistic regression for tumor IHC marker as the dependent variable, with a negative staining of the tumor marker as reference category. If adjusted OR are not given, there is not enough power to perform adjusted regression analyses. ^ǂ^ Women with diabetes treated with insulin analogues regardless the use of concomitant human insulin (n=22) or noninsulin antidiabetic drugs. ^¥^ Women with diabetes treated with human insulin only regardless the use of concomitant noninsulin antidiabetic drugs. ^§^ Women with diabetes treated with insulin (analogues) regardless the use of concomitant noninsulin antidiabetic drugs. ^†^ Women with diabetes treated only with diet and exercise and users of noninsulin antidiabetic drugs only. ^#^ Adjusted for menopause (pre/post) at breast cancer diagnosis and BMI closest measure prior to breast cancer diagnosis (continuous). Cases and controls were matched on age at breast cancer diagnosis. *p-ER= phosphorylated estrogen receptor, EGFR=epidermal growth =factor receptor, p-ERK= phosphorylated extracellular signal-regulated kinase, p-mTOR=phosphorylated mechanistic target of rapamycin, IGF1R=insulin growth factor 1 receptor, OR=Odds Ratio, CI=Confidence Interval.*

.
