# Supplementary material for: The association of diabetes mellitus and insulin treatment with expression of insulin-related proteins in breast tumors
Source: BMC Cancer. 2018 Feb 27;18:224. doi: 10.1186/s12885-018-4072-8 (PMC6389252; doi:10.1186/s12885-018-4072-8)
Supplement: Supplementary file 2 — Document 1. REMARK checklist. (PDF 68 kb) [file 12885_2018_4072_MOESM2_ESM.pdf]

BioMed Central
The Open Access Publisher

PRE-CHECK CHECKLIST

Pass

MANUSCRIPT TITLE The association of diabetes mellitus and insulin treatment with expression of insulin-related proteins in breast tumours.

AUTHORS Heleen K Bronsveld, Marie L De Bruin, Jelle Wesseling, Joyce Sanders, Ingrid Hofland, Vibeke Jensen, Marloes T Bazelier, Bas ter Braak, Anthonius de Boer, Peter Vestergaard, Marjanka Schmidt

REPORT DATE Feb 16 2017, 13:56 (UTC -0500)

AUTHORSHIP

- ✔ All authors have provided unique email addresses in the submission data or manuscript, and the corresponding author email has been verified
- ✔ Potential and perceived conflicts of interest have been declared
- ✔ Funding sources have been disclosed

ARTICLE BODY

- ✔ Manuscript contains a clear and meaningful research question and provides rationale for the study
- ✔ English language is of sufficient quality to allow for review
- ✔ Text has not been published elsewhere
- ✔ Manuscript does not contain text that may be perceived as defamatory

FIGURES

- ✔ Gel/blot images and cell images have not been noticeably manipulated or duplicated
- ✔ Figures/Tables/Supplemental Items do not contain any copyrighted material or patient identifying information
- ✔ Figures/Tables/Supplemental Items have all been referenced in the text

APPROVALS AND PERMISSIONS

- ✔ Human subjects research has ethical approval and informed consent
- ☐ Clinical trials are registered and there is a real record
- ☐ Animal models have ethical approval
- ☐ Field samples have permission for use and ethical approval
- ☐ Third party data have permission for use or are publicly available

REQUESTED CHANGES

No changes requested; the journal editor may request additional information at a later time.

Details

AUTHORSHIP

- The email for corresponding author Marjanka K Schmidt matches an institutional website for this author. All other authors have provided unique email addresses in the manuscript or submission data.
- The manuscript and supporting information were checked for a COI statement. The authors state that they have no conflicts of interest. All authors have hospital/university affiliations. No obvious COIs are noted.
- Manuscript was searched for a statement regarding funding. Funding is disclosed in the Declarations/Funding section.

ARTICLE BODY

- The manuscript was checked for a clear research statement (objectives/aims). A valid research question/objective is stated and a rationale for the study is clear.
- The English language was spot checked throughout the manuscript. The grammar, word choice, and punctuation are of sufficient quality to allow for peer review.
- iThenticate report was checked for substantial overlap between the abstract, introduction, results, and discussion of the manuscript and other published sources. There are no concerns regarding text duplication for this manuscript.
- Manuscript text was scanned for potentially defamatory content regarding any person/group/company/organization/product. No potentially defamatory text was identified.

FIGURES

- Cell images were enlarged and visually inspected for possible digital manipulation by changing brightness and contrast levels. Images were also visually inspected for possible duplication within the manuscript. No items of concern were found.
- Figures, tables, and supplemental information were checked for items that appeared to require copyright approval and for items that disclose patient identifying information. There were no items of concern.
- The text was checked for figure, table, and supplemental item references. All included items are referenced and there are no references that point to absent items.

APPROVALS AND PERMISSIONS

- The manuscript was checked for a statement regarding ethical approval by a named committee and for a statement regarding human subject consent. These statements are present and adequate.

Research Square is dedicated to improving the way discoveries are shared. We are a growing team of scientists, researchers, language experts, software developers, and publishing industry veterans working together to find new ways to help researchers succeed. ResearchSquare.com
