# Supplementary figures and images for: The association of diabetes mellitus and insulin treatment with expression of insulin-related proteins in breast tumors
Source: BMC Cancer. 2018 Feb 27;18:224. doi: 10.1186/s12885-018-4072-8 (PMC6389252; doi:10.1186/s12885-018-4072-8)

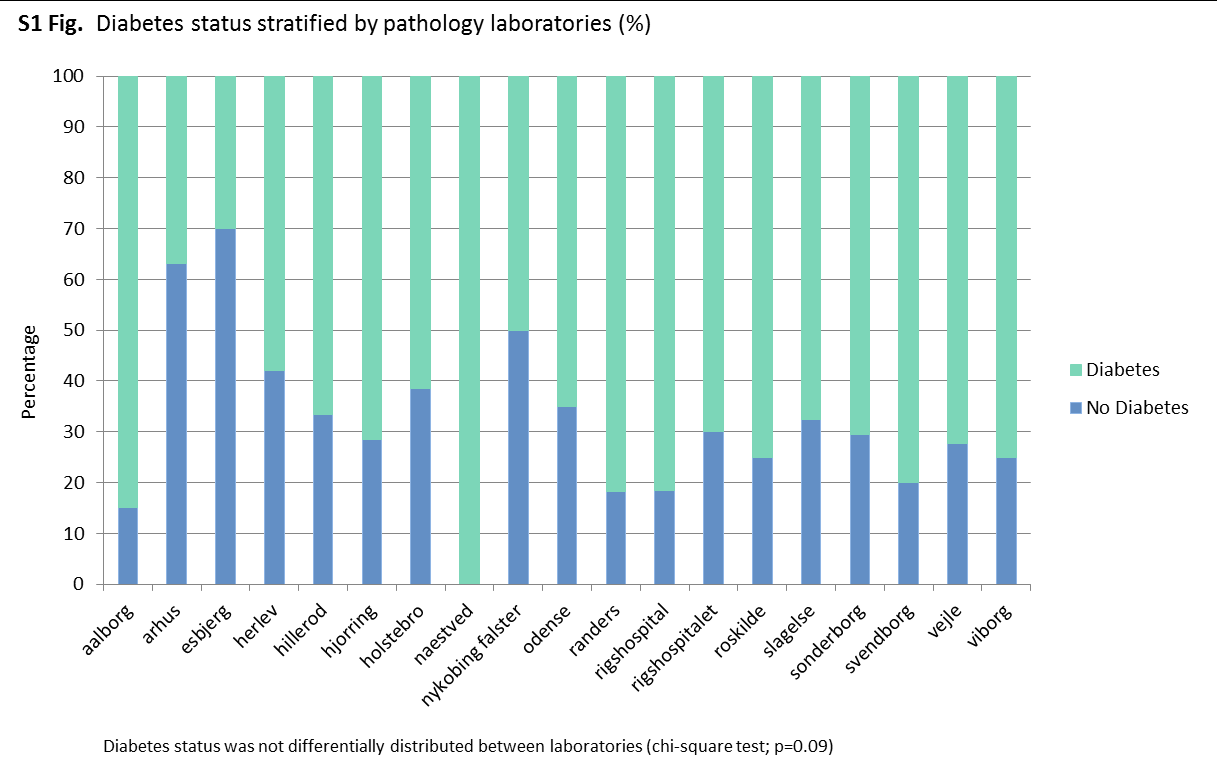

Supplement: Supplementary file 3 — Diabetes status stratified by pathology laboratories (%). (PNG 38 kb) [file 12885_2018_4072_MOESM3_ESM.png]
